# Processing-Structure-Properties Relationships of Glycerol-Plasticized Silk Films

**DOI:** 10.3390/molecules27041339

**Published:** 2022-02-16

**Authors:** Hao Lyu, Ziyang Sun, Yang Liu, Xin Yu, Chengchen Guo

**Affiliations:** 1 School of Materials Science and Engineering, Zhejiang University, Hangzhou 310027, China; lyuhao@westlake.edu.cn; 2 School of Engineering, Westlake University, Hangzhou 310024, China; sunziyang@westlake.edu.cn; 3 Institute of Biomedical Engineering and Health Sciences, Changzhou University, Changzhou 213164, China; liuyang@cczu.edu.cn

**Keywords:** silk film, glycerol, molecular structures, mechanical properties

## Abstract

Silk possesses excellent mechanical properties and biocompatibility due to its unique protein sequences and hierarchical structures. Thus, it has been widely used as a biomaterial in a broad spectrum of biomedical applications. In this study, an in-depth investigation of glycerol-plasticized silk films was carried out to understand the processing-structure-properties relationships. A series of glycerol-plasticized silk films with glycerol contents in the range of 0 to 30% (*w*/*w*) were prepared. The molecular structures and organizations of silk proteins and the interactions between glycerol and proteins were studied using FTIR, XRD, and DSC. At a low glycerol content (<12%), DSC revealed that the glass transition temperature and thermally induced crystallization temperature decreased as the glycerol content increased, implying that glycerol mainly interacts with silk proteins through hydrogen bonding. As the glycerol content further increased, the chain mobility of the silk proteins was promoted, leading to the formation of β-sheet structures, water insolubility, and increased crystallinity. In addition, the stretchability and toughness of the films were significantly enhanced. The role of glycerol as a plasticizer in regulating the silk protein structures and determining the properties of the films was thoroughly discussed.

## 1. Introduction

With the global trend of achieving carbon neutrality to mitigate the effects of climate change, the promotion of natural protein-based materials as an alternative to petroleum-based materials is attracting increasing attention in the food, healthcare, and packaging industries [1,2,3,4]. Silk proteins derived from silkworm cocoons have been studied and widely used in recent years due to their excellent mechanical properties, good biocompatibility, and aqueous-based preparation process [5,6,7,8,9,10,11,12]. Silk proteins can be processed into different materials such as films, hydrogels, and sponges to satisfy the needs of various applications [13,14,15,16]. Regarding silk material processing, some plasticizers, including polyols and lipids, have been used to tune the physical properties of the materials, where the plasticization leads to structural changes of the materials and softening of the brittle materials in the glass state [17,18,19]. Previous studies have shown that glycerol is an excellent plasticizer to regulate the protein structures and mechanical properties of silk materials. Specifically, the addition of glycerol can induce β-sheet structures and increase the stretchability of the silk films [20,21,22]. Brown et al. proposed that glycerol may simultaneously induce and interfere with β-sheet formation in silk materials, causing some improper folding that results in less-organized silk II structures even after the glycerol is removed [22]. These studies provide some understanding of the plasticization effect of glycerol in silk materials, as well as the interactions between glycerol and silk proteins. However, a clear understanding of processing-structure-properties relationships is still lacking, particularly for the glycerol-plasticized silk film with a relatively low glycerol content (<12%).

In this study, an in-depth investigation of glycerol-plasticized silk films was carried out to understand the processing-structure-properties relationships. We prepared a series of glycerol-plasticized silk films with glycerol contents ranging from 0 to 30%. Then, we characterized these films using Fourier-transform infrared (FTIR) spectroscopy, X-ray diffraction (XRD), differential scanning calorimetry (DSC), and mechanical testing to understand the influence of glycerol on the protein structures and organizations, thermal property, water solubility, and mechanical properties. Specifically, FTIR and XRD were used to investigate the protein structures at the molecular level and probe the interactions between protein and glycerol. DSC was used to study the glass transition and thermally induced crystallization of the materials. The results showed that at a low glycerol content (<12%), the glass transition temperature, and thermally induced crystallization temperature of the films decreased as the glycerol content increased, implying that glycerol mainly interacts with silk protein chains through hydrogen bonding. As the glycerol content further increased, the mobility of silk protein chains was promoted, inducing the formation of β-sheet structures and increased crystallinity. In addition, the stretchability and toughness of the films were significantly enhanced. At a high glycerol content (>22%), glycerol started to become excessive, and the excessive glycerol did not influence the structure of silk. Based on the experimental results, the role of glycerol as a plasticizer in regulating the silk protein structures and determining the properties of the films was thoroughly discussed. This study provides a solid theoretical basis for fabricating glycerol-plasticized silk materials with controlled structures and properties. These materials show great potential in flexible electronics, bioplastics, and medical devices.

## 2. Materials and Methods

### 2.1. Chemicals and Materials

Sodium carbonate (Na_2_CO_3_, 99.5%), lithium bromide (LiBr, 99%), and glycerol (99%) were purchased from Aladdin Industrial Corporation (Shanghai, China). Silkworm cocoons (*Bombyx mori*) were purchased from a local store in Hangzhou, China. Dialysis tubing was purchased from Solarbio Science & Technology Co., Ltd. (Beijing, China).

### 2.2. Preparation of Regenerated Silk Solution

The regenerated silk solution was prepared following the reported method [15]. Briefly, the cocoons were cut into small pieces and boiled for 30 min in a solution of 0.02 M Na_2_CO_3_ to remove sericin (degumming). The cleaned and dried degummed silk fibers were dissolved in the 9.3 M LiBr aqueous solution (20% wt/vol) at 60 °C for 4 h. The silk solution was then dialyzed against deionized water (DI water) for 72 h to remove salt ions (MWCO 3500, Solarbio). The water was changed after 1 h, 4 h, 8 h, 24 h, 36 h, 48 h, and 60 h. After dialysis, the solution was purified by centrifugation twice (9000 rpm, 20 min) to remove impurities. The concentration of the purified silk solution was determined to be ~6 wt% (*w*/*v*) and the purified silk solution was stored in a 4 °C refrigerator for further use.

### 2.3. Preparation of Glycerol-Plasticized Silk Films

The glycerol-plasticized silk films were prepared by a solution casting method. Briefly, 100 mg/mL glycerol solution was prepared in advance. Then, specific amounts of the glycerol solution were added into different batches of silk solution (~6 wt%) respectively for preparing a series of glycerol-plasticized silk films with different glycerol contents (2%, 4%, 6%, 8%, 10%, 12%, 14%, 16%, 18%, 20%, 22%, 24%, 26%, 28%, 30%, *w*/*w*). For each condition, 3.5 mL of the mixed solution was transferred into a petri-dish with a diameter of 60 mm, followed by drying at 25 °C and 60% relative humidity (RH) for 24 h to obtain the dried film. The thickness of the dried silk films was around 50 μm. In this work, Gly-X was used to annotate the glycerol-plasticized silk films where X refers to the glycerol content (X%) in the films. After drying, the films were stored in a vacuum drier to avoid humidity. The characterization tests were performed instantly following film fabrication.

### 2.4. Fourier-Transform Infrared (FTIR) Spectroscopy

The molecular structures of the glycerol-plasticized silk films were investigated by FTIR spectroscopy on a Bruker ALPHA Ⅱ FTIR spectrometer equipped with an attenuated total reflection (ATR) attachment (with a diamond crystal plate). For each type of silk film, at least three spectra were collected with a resolution of 4 cm^−1^ and 64 scans. The peak devolution of amide I region was performed using a home-developed python package. The Gaussian peaks at 1620 cm^−1^ and 1698 cm^−1^ were assigned to β-sheet structure while the Gaussian peaks at 1645 cm^−1^ and 1685 cm^−1^ were assigned to random coil/α-helix and β-turn structures, respectively. The ratios of different secondary structures were then estimated from the deconvolution.

### 2.5. Thermal Analysis

The thermal properties of the glycerol-plasticized silk films were characterized by differential scanning calorimetry (DSC) on a DSC3 instrument (Mettler Toledo, Greifensee, Switzerland) under a dry nitrogen gas flow of 50 mL/min. 3–8 mg of each sample was loaded into a 40 μL aluminum pan for measurement. The samples were heated from 0 to 250 °C with a heating rate of 10 °C/min. For glass transition temperature (*T_g_*) measurement, two heating scans were applied. The first heating scan was from 20 to 200 °C with a heating rate of 20 °C/min for removing free water in the films and the second heating scan was from −30 to 220 °C with a heating rate of 20 °C/min.

### 2.6. X-ray Diffraction (XRD)

The XRD profiles of the glycerol-plasticized silk films were collected on a X-ray Diffractometer (Bruker, D8 Advance, Karlsruhe, Germany) according to previous reports [23,24]. Each sample was characterized with a tube voltage of 40 kV, a current of 40 mA, and a plate rotational speed of 5 rad/min. The wavelength (λ) of X-ray was 1.54 Å and the diffraction angle (2θ) was recorded from 5° to 60°. The results were analyzed and deconvoluted to determine the crystallinity.

### 2.7. Water Solubility

The water solubility of the glycerol-plasticized silk films was evaluated by UV-Vis spectrophotometry due to the absorption of the tyrosine residue in the silk proteins (~280 nm) [21]. Briefly, the silk films were cut into approximately 10 × 10 mm^2^ pieces and weighed. The mass was noted as *m*_1_. The film pieces were then incubated in 10 mL DI water at 37 °C with a shaking rate of 50 rpm for 24 h. After incubation, the film pieces were dried in an oven and weighed. The mass was noted as *m*_2_. The residual mass ratio of the films was calculated by the following equation:

(1) $$\mathrm{residual}\;\mathrm{mass}\;\mathrm{ratio}=\frac{m_{2}}{m_{1}}\times 100\%$$

The remaining solution was characterized by UV-vis spectroscopy to obtain the absorption intensity at 280 nm, which can be used to estimate the dissolution ratio of the films. The standard curve of absorption was obtained using pure silk solutions with various known concentrations.

### 2.8. Mechanical Test

The mechanical properties of the glycerol-plasticized silk films were characterized by tensile testing on a universal testing machine (Cellscale UniVert, Waterloo, Ontario, Canada) equipped with a 50 N load cell. Before testing, each sample was cut into scaled dumbbell-shaped strips according to ASTM standard D412-A. During the test, a controlled strain rate of 20 mm/min was applied to all samples, and the data were collected with 15 Hz. For each type of silk film, at least six measurements were tested and reported as the mean ± standard deviation. All measurements were tested at room temperature and 55~60% humidity. Ultimate strength was determined as the highest stress value on the stress-strain curve, and the strain to failure was the last point before a >10% decrease of stress. The Young’s modulus was determined by calculating the slope of stress-strain curve at the elastic deformation range. The toughness was determined by calculating the integral area under the stress-strain curve.

### 2.9. Statistics

All replicate experiments were performed with a minimum of N = 3 for each data point. The results were performed by SPSS (19.0) using a one-way analysis of variance (ANOVA). Significant differences were defined when *p* < 0.05 and very significant differences when *p* < 0.01.

## 3. Result and Discussion

### 3.1. Molecular Structures of the Glycerol-Plasticized Silk Films

**Figure 1:**
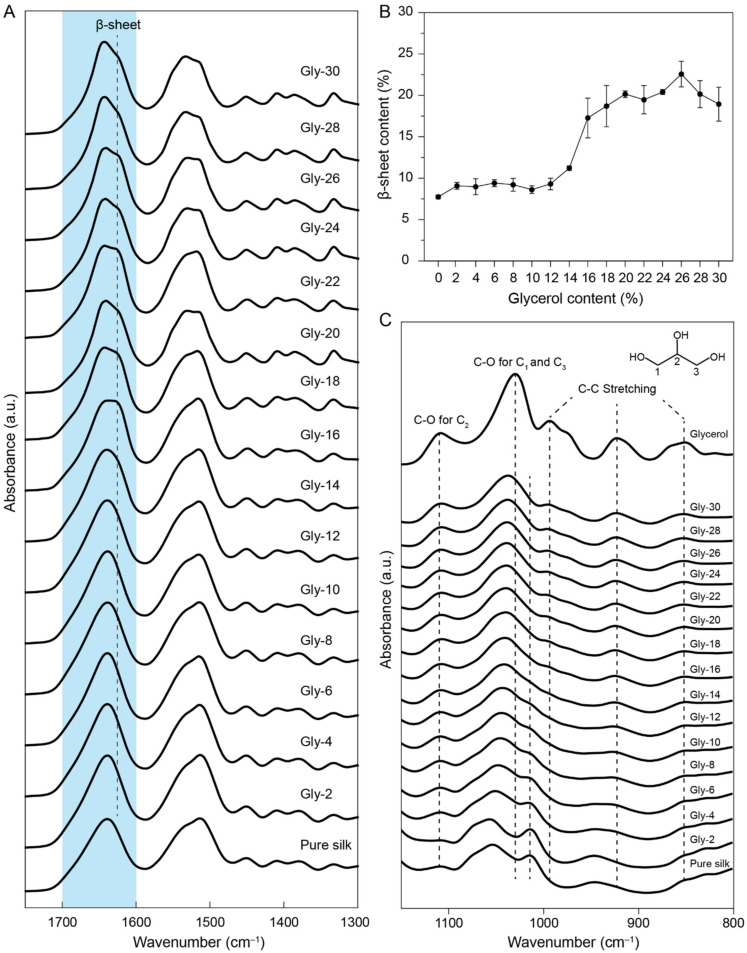
(**A**) FTIR spectra of the glycerol-plasticized silk films with highlighted amide Ⅰ region. (**B**) β-sheet contents of the glycerol-plasticized silk films estimated from the deconvolution of the amide I region. Three FTIR spectra were collected for each sample and spectral deconvolution was performed to estimate the β-sheet contents. (**C**) FTIR spectra of the glycerol-plasticized silk films and glycerol with a spectral region of 800–1150 cm^−1^.

The molecular structures of the glycerol-plasticized silk films were characterized by FTIR spectroscopy and X-ray diffraction. Figure 1A shows the FTIR spectra of the films and the amide I region (1600–1800 cm^−1^) was highlighted in blue. Since glycerol has no characteristic absorbances in the amide I region (Figure S1), the deconvolution analysis of the amide Ⅰ region was performed to estimate the β-sheet contents of the films. The result is shown in Figure 1B and the details of the deconvolution were summarized in Figure S2. When the glycerol content in the film was less than 12%, no significant number of β-sheet structures was found (less than 10%). The result indicated that introducing a small amount of glycerol did not significantly influence the secondary structures of silk proteins in the films. When the glycerol content was increased to 12%, the band at 1620 cm^−1^ assigned to β-sheet structures was enhanced, indicating that more β-sheet structures were formed in the films. Particularly, when the glycerol content reached 20%, a β-sheet content of ~20% was found. When the glycerol content was higher than 18%, the β-sheet content in the films was in the range of 20–23%, indicating that further addition of glycerol beyond 18% could not introduce more β-sheet structures.

To better understand the molecular interactions between glycerol and silk proteins, we analyzed the IR spectra with a spectral region within 800–1150 cm^−1^ (Figure 1C). For pure glycerol, the absorbances at 850 cm^−1^, 925 cm^−1^, and 995 cm^−1^ were assigned to the C-C stretching while the absorbances at 1030 cm^−1^ and 1108 cm^−1^ were assigned to the C_1, 3_-O and C_2_-O respectively (The designations of C_1_,C_2_, and C_3_ were listed in Figure 1C) [25,26,27]. For the pure silk film, the absorbances were assigned to the vibrations of the side chains in amino acid residues. The absorbances in the range at ~1000–1100 cm^−1^ were assigned to the C-O stretching of the serine and threonine residues [28]. As the glycerol content in the film increased, the absorbance at 1014 cm^−1^ decreased and the absorbance at 1055 cm^−1^ shifted. This result indicated that the glycerol molecules interact with the hydroxyl groups in the serine and threonine residues via hydrogen bonding. From the spectra, it was also found that some free glycerol may present in the materials at high glycerol contents (>18%) since the characteristic absorbances of glycerol were clearly observed.

**Figure 2:**
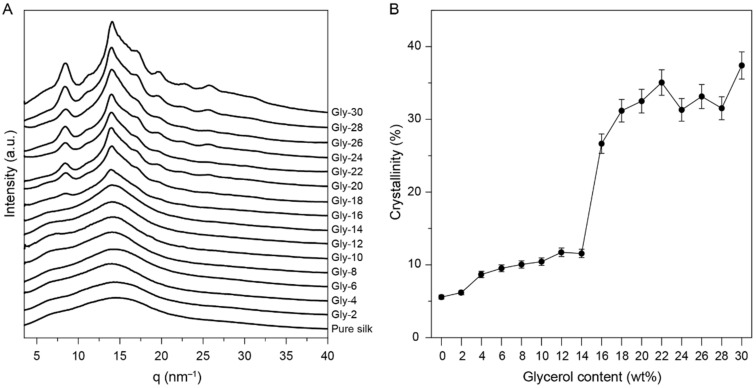
(**A**) XRD profiles of the silk and the glycerol-plasticized silk films. (**B**) Degree of crystallinities of the glycerol-plasticized silk films calculated from the devolution of XRD profiles. An error of ±5% was indicated for each sample due to peak deconvolution.

X-ray diffraction (XRD) was used to characterize the crystalline state of the glycerol-plasticized silk films, and the results are shown in Figure 2. At low glycerol contents (<12%), the plasticized silk films showed similar broad peaks as the pure silk film, which indicated that the materials predominantly present amorphous structures with low crystallinities. At a glycerol content of 16%, sharp peaks with q values of 8.5 and 14.1 nm^−1^ were detected (Figure 2A). With further increase of the glycerol content, more peaks were detected. These results indicated that the crystallization was greatly induced at a glycerol content over 16%. The deconvolution of the XRD profiles were performed to estimate the crystallinities of the glycerol-plasticized silk films (Figure 2B and Figure S3). Briefly, the peaks used in the deconvolution include crystalline components (6.6 nm^−1^, 8.5 nm^−1^, 14.1 nm^−1^, 14.7 nm^−1^, 17.3 nm^−1^, 19.8 nm^−1^, 22.7 nm^−1^, 26 nm^−1^, 28.1 nm^−1^, 30.5 nm^−1^) and amorphous components (~15.0 nm^−1^, ~28.0 nm^−1^). The deconvolution results showed that the crystallinities of the films were ~10% at low glycerol contents (<12%). However, a significant increase of crystallinity was observed for the films containing 16% glycerol, showing a crystallinity of 27 %. At higher glycerol contents (>16%), the crystallinities of the films were primarily in the range of 30–35%. These results agreed well with the FTIR results, demonstrating the significant change of molecular structures at a glycerol content ~14–18%. Based on the FTIR and XRD results, the molecular structures of the silk proteins and the interactions between glycerol and the silk proteins during the film fabrication process can be partially elucidated. At a low glycerol content (<12%), glycerol molecules primarily interact with the hydroxyl groups in the silk proteins via hydrogen bonding, leading to the disruption of the intermolecular interactions between protein chains and the promotion of the chain mobility (plasticization) in the amorphous domains. Such plasticization is not sufficient to promote the formation of β-sheet structures during the drying process (25 °C, RH 60%) until the glycerol content reaches ~14%. As the glycerol content surpasses 14%, more β-sheet structures are induced, but remain less compared to the silk films treated with methanol [29]. XRD results show that both the silk I and silk II structures are present in the films where the silk I structure is predominant [30]. It is known that the silk I structure is composed primarily of helical or turn structures [29,30], so the overall β-sheet contents for the films with over 14% glycerol are relatively less compared with silk materials presenting predominate silk II structure.

### 3.2. Thermal Properties of the Glycerol-Plasticized Silk Films

**Figure 3:**
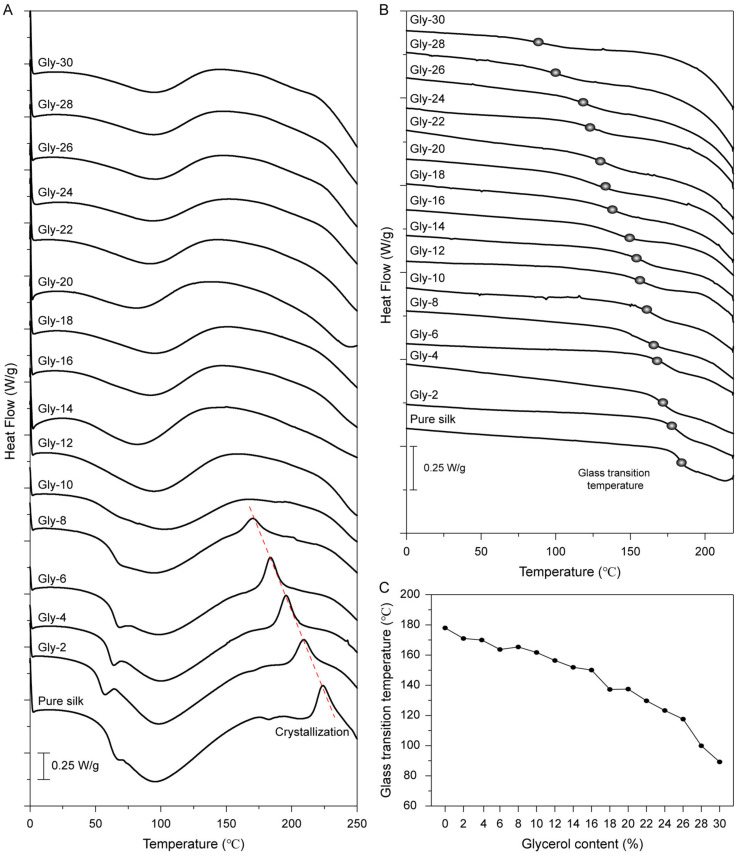
(**A**) DSC curves of glycerol-plasticized silk films with various glycerol contents. The samples were heated from 0 to 250 °C with a heating rate of 10 °C/min. (**B**) The second scan DSC curves of glycerol-plasticized silk films with various glycerol contents. The first heating scan was from 20 to 200 °C with a heating rate of 20 °C/min for removing free water in the films and the second heating scan was from −30 to 220 °C with a heating rate of 20 °C/min. (**C**) Glass transition temperatures of glycerol-plasticized silk films with various glycerol contents.

The thermal properties of the glycerol-plasticized silk films were investigated by DSC, and the results are shown in Figure 3. In Figure 3A, a water-associated glass transition temperature (*T_g_′*) was clearly observed around 65 °C for the silk film with a glycerol content below 10%. The broad endothermic peak detected between 40 and 160 °C was attributed to the removal of water from the films. For the pure silk film, a crystallization peak was observed at around 220 °C. Interestingly, the position of the crystallization peak shifted to lower values as the glycerol content increased up to 10%. This result indicated that glycerol could promote chain mobility and reduce the energy barrier for the thermally induced crystallization in silk films. Upon the glycerol content reaching 10% or above, the crystallization could no longer be detected. This may be due to the partial crystallization of the silk proteins during the fabrication at room temperature, as confirmed by XRD results (Figure 2).

After the removal of water, a glass transition temperature (*T_g_*) of 178 °C was observed in the second scan of DSC curves for the pure silk film (Figure 3B), which is consistent with previous reports [31,32]. As the glycerol content increased, the *T_g_* of the films presented a decreasing trend, indicating the increase of the mobility of the silk protein chains in the amorphous domains. The glycerol molecules penetrate the amorphous domains and thus decrease the interactions between silk protein chains. As shown in Figure 3C, the *T_g_* of the film with glycerol content of 30% was detected around 90 °C, which is significantly lower than the *T_g_* of the pure silk film.

### 3.3. Water Solubility of the Glycerol-Plasticized Silk Films

**Figure 4:**
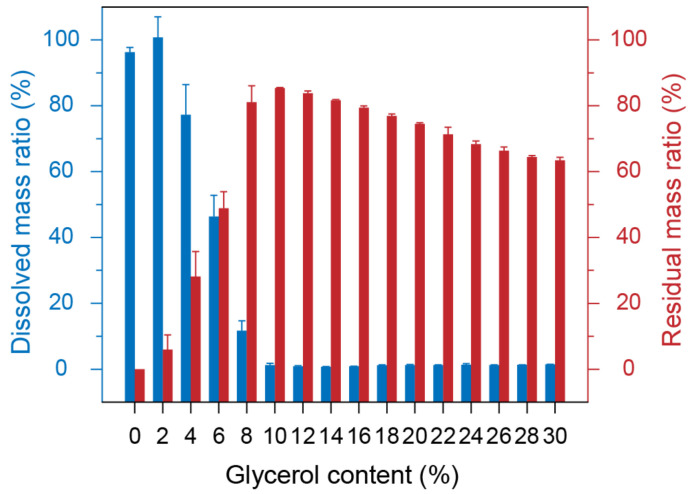
Water solubility of the glycerol-plasticized silk films.

The water solubility of the glycerol-plasticized silk films was characterized by UV-vis spectroscopy. Tyrosine residue in silk proteins has a characteristic absorption peak at 280 nm while glycerol has no obvious absorption in this region [21]. Figure 4 shows the water solubility and the residual mass of the glycerol-plasticized silk films. It is shown that the pure silk film and Gly-2 film could be dissolved in water completely [33]. However, the solubility of the films in water reduced dramatically as the glycerol content increased up to 10%. The films became water-insoluble when the glycerol content was greater than 10%. This result can be interpretated by the crystallization induced by the addition of glycerol as reflected by XRD and DSC results (Figure 2 and Figure 3). In addition, it was found that glycerol was completely diffused out of all blend films after incubation in water since the sum of the residual mass and the glycerol content was close to 100%.

### 3.4. Mechanical Properties of the Glycerol-Plasticized Silk Films

**Figure 5:**
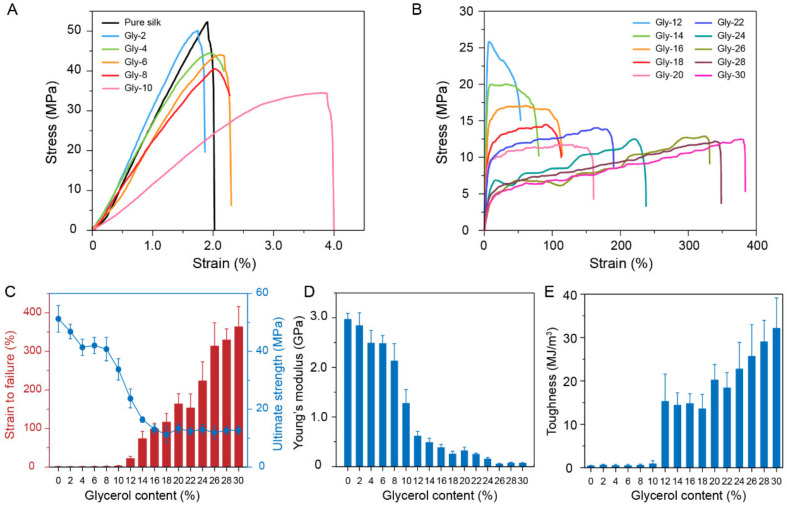
(**A**,**B**) Representative strain-stress curves of the glycerol-plasticized silk films. (**C**) The failure strains and the ultimate strengths of the glycerol-plasticized silk films. The Young’s modulus (**D**) and the toughness (**E**) of the glycerol-plasticized silk films.

The mechanical properties of the glycerol-plasticized silk films were characterized by tensile testing. The representative strain-stress curves of the films are shown in Figure 5 and the results were summarized in Table 1. At low glycerol contents (<12%), the films were brittle with failure strains less than 3% and Young’s modulus around 2.5 GPa (Figure 5C,D) [23,34]. At this stage, glycerol is not enough to effectively disrupt the interactions between silk protein chains, leading to the limited promotion of the chain mobility. As the glycerol content increased over 12%, the films showed distinct plastic deformation (Figure 5B), indicating the enhancement of the chain mobility. The failure strain increased up to around 360% at the glycerol content of 30%. This result indicated that the glycerol located in the amorphous domains could enhance the mobility of silk protein chains effectively when the glycerol content was greater than 12%. Regarding the ultimate strength, it showed a clear decrease from 51.26 ± 4.61 MPa to 13.10 ± 1.50 MPa as the glycerol content increased from 0 to 16%. However, the ultimate strength did not decrease significantly as the glycerol content continued to increase. This trend is due to the dynamic interactions between the silk proteins and the glycerol, and the conformation change of the silk proteins. Specifically, at a low glycerol content, glycerol is capable of penetrating silk protein chains and breaking the initial hydrogen bonds formed between the silk protein chains (plasticization), resulting in the decrease of ultimate strength. However, as the glycerol content increased over 16%, the crystallinity of the films increased correspondingly and reached a plateau. Meanwhile, the glycerol for interfering the hydrogen bonding between the silk protein chains was saturated in the amorphous domains of the materials. Therefore, the ultimate strength is primarily determined by the crystallinity of the film at a relatively higher glycerol content. The Young’s modulus of the films showed a decreasing trend as the glycerol content increased (Figure 5D). However, the toughness of the films showed significant increase at a glycerol content of 12% (Figure 5E), which is attributed to the increase of failure strain. At this point, the plasticity of the films was greatly enhanced.

### 3.5. The Role of Glycerol in Plasticizing Silk Materials

**Figure 6:**
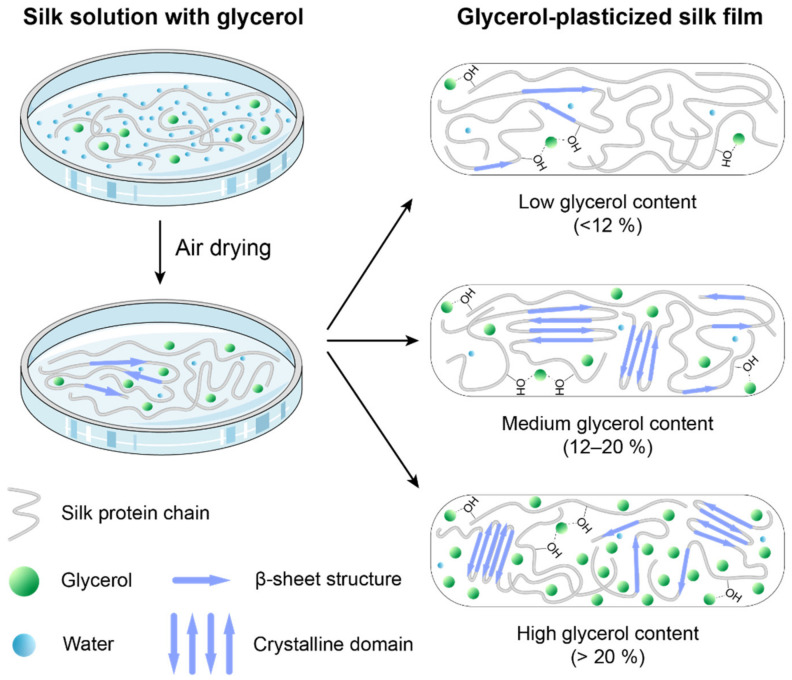
A schematic illustration of the role of glycerol in plasticizing silk proteins during the film fabrication.

During the fabrication process of the glycerol-plasticized silk films, glycerol plays a critical role in regulating the structures of the silk proteins. Glycerol has a much lower vapor pressure than water at room temperature. Therefore, during the drying process, a very limited amount of glycerol evaporates. In this work, it is found that glycerol has a significant effect on the molecular structures of silk proteins during the drying process depending on the content. The role of glycerol in plasticized films can be summarized as follows (Figure 6). At a low glycerol content (<12%), glycerol molecules primarily interact with the hydroxyl groups in the silk proteins via hydrogen bonding [22], resulting the disruption of the intermolecular interactions between the protein chains and the promotion of the chain mobility in the amorphous domains. Such plasticization is not sufficient to induce the transformation of silk secondary structure from amorphous to β-sheet structures but will induce limited crystallization. The films are brittle with decreasing strength and Young’s modulus as the glycerol content increases. At a medium glycerol content (12–20%), the mobility of the silk protein chains is enhanced significantly, leading to the formation of β-sheet structures. The formed β-sheet structures further induce the formation of ordered crystalline domains in the films. As a result, the films become completely water insoluble. The increased mobility of silk protein chains in the amorphous domains results in increased stretchability and toughness. At a high glycerol content (>22%), the β-sheet content and crystallinity are not changed significantly. The increased amount of glycerol present in the amorphous domains further promotes the mobility of the silk protein chains. Therefore, the *T_g_* of the plasticized films shows a continuously decreasing trend. It must be noted that a small amount of residual water is present besides glycerol in the dry silk films. The residual water is also considered as a plasticizer that influences the properties of the silk films [31,34,35]. Therefore, precise control of the drying conditions (temperature, humidity) is critical for obtaining silk films with designed structures and properties.

## 4. Conclusions

In this study, we prepared glycerol-plasticized silk films with glycerol contents ranging from 0 to 30% and investigated the processing-structure-properties relationships. It is found that the glycerol molecules mainly interact with the silk proteins via hydrogen bonding without inducing significant structural transitions at a low glycerol content (<12%). Additionally, both the glass transition temperature and thermally induced crystallization temperature decrease with increasing the glycerol content. As the glycerol content further increased, the chain mobility of the silk proteins was promoted, leading to the formation of β-sheet structures, water insolubility, and increased crystallinity. In addition, the stretchability and toughness of the films are significantly enhanced. When the glycerol content is over 20%, the glycerol in the films has little influence on the molecular structures of the silk proteins. However, the stretchability and toughness of the films further increase due to the promotion of the protein chain mobility in the amorphous domains. The comprehensive understanding of the processing-structure-properties relationships in glycerol-plasticized silk films will facilitate the development of silk materials with controlled structures and properties for flexible electronics, bioplastics, and medical devices.

## Figures and Tables

**Table 1:** Summary of the mechanical properties for the glycerol-plasticized silk films.

Glycerol Content (%)	Young’s Modulus (GPa)	Strain to Failure (%)	Ultimate Strength (MPa)	Toughness (MJ/m^3^)
**0**	2.97 ± 0.12	1.69 ± 0.22	51.26 ± 4.61	0.46 ± 0.09
**2**	2.85 ± 0.25	1.80 ± 0.09	46.86 ± 2.48	0.65 ± 0.15
**4**	2.49 ± 0.25	1.90 ± 0.21	41.42 ± 2.80	0.53 ± 0.22
**6**	2.48 ± 0.16	2.02 ± 0.36	42.04 ± 2.80	0.55 ± 0.19
**8**	2.13 ± 0.35	2.22 ± 0.56	40.81 ± 4.08	0.64 ± 0.17
**10**	1.28 ± 0.27	3.39 ± 0.91	33.81 ± 3.66	0.93 ± 0.69
**12**	0.62 ± 0.09	22.9 ± 4.92	23.71 ± 3.24	15.34 ± 6.22
**14**	0.49 ± 0.08	74.24 ± 18.68	16.42 ± 0.91	14.45 ± 2.79
**16**	0.39 ± 0.06	99.83 ± 13.59	13.10 ± 1.50	14.84 ± 2.19
**18**	0.26 ± 0.05	117.18 ± 21.97	11.18 ± 2.67	13.61 ± 3.27
**20**	0.32 ± 0.07	164.56 ± 25.65	13.37 ± 1.52	20.26 ± 3.51
**22**	0.25 ± 0.02	153.62 ± 36.33	12.38 ± 1.33	18.45 ± 3.47
**24**	0.16 ± 0.03	223.89 ± 49.13	13.03 ± 1.75	22.81 ± 6.06
**26**	0.05 ± 0.01	314.4 ± 59.72	11.94 ± 2.21	25.75 ± 7.22
**28**	0.08 ± 0.01	330.34 ± 28.18	12.68 ± 1.33	29.08 ± 4.87
**30**	0.07 ± 0.01	364.42 ± 51.83	12.74 ± 1.51	32.19 ± 6.96

## Data Availability

Data are included in the text; raw data are available from the corresponding authors.

## Supplementary Materials

The following are available online, Figure S1: FTIR spectra of pure glycerol in the range of 800–1800 cm^−1^, Figure S2: The deconvolution of the amide I region for glycerol-plasticized silk films, Figure S3: The deconvolution and peak fitting process of XRD curve for different silk-glycerol films, Figure S4: Statistical analysis for β-sheet contents of the glycerol-plasticized silk films.

## Sample Availability

Samples of the tested materials and compounds are not available from the authors.

## References

[1] Torculas M., Medina J., Xue W., Hu X. (2016). Protein-based bioelectronics. ACS Biomater. Sci. Eng..

[2] Karan H., Funk C., Grabert M., Oey M., Hankamer B. (2019). Green bioplastics as part of a circular bioeconomy. Trends Plant Sci..

[3] Mohamed S.A., El-Sakhawy M., El-Sakhawy M.A.-M. (2020). Polysaccharides, protein and lipid-based natural edible films in food packaging: A review. Carbohydr. Polym..

[4] Kapoor S., Kundu S.C. (2016). Silk protein-based hydrogels: Promising advanced materials for biomedical applications. Acta Biomater..

[5] Vepari C., Kaplan D.L. (2007). Silk as a biomaterial. Prog. Polym. Sci..

[6] Yarger J.L., Cherry B.R., Van Der Vaart A. (2018). Uncovering the structure–function relationship in spider silk. Nat. Rev. Mater..

[7] Guo C., Li C., Mu X., Kaplan D.L. (2020). Engineering Silk Materials: From Natural Spinning to Artificial Processing. Appl. Phys. Rev..

[8] Guo C., Zhang J., Jordan J.S., Wang X., Henning R.W., Yarger J.L. (2018). Structural Comparison of Various Silkworm Silks: An Insight into the Structure-Property Relationship. Biomacromolecules.

[9] Guo C., Zhang J., Wang X., Nguyen A.T., Liu X.Y., Kaplan D.L. (2017). Comparative Study of Strain-Dependent Structural Changes of Silkworm Silks: Insight into the Structural Origin of Strain-Stiffening. Small.

[10] Li C., Guo C., Fitzpatrick V., Ibrahim A., Zwierstra M.J., Hanna P., Lechtig A., Nazarian A., Lin S.J., Kaplan D.L. (2019). Design of biodegradable, implantable devices towards clinical translation. Nat. Rev. Mater..

[11] Mu X., Wang Y., Guo C., Li Y., Ling S., Huang W., Cebe P., Hsu H.H., De Ferrari F., Jiang X. (2020). 3D Printing of Silk Protein Structures by Aqueous Solvent-Directed Molecular Assembly. Macromol. Biosci..

[12] Wang X., Guo C., Chen Y., Tozzi L., Szymkowiak S., Li C., Kaplan D.L. (2020). Developing a self-organized tubulogenesis model of human renal proximal tubular epithelial cells in vitro. J. Biomed. Mater. Res. A.

[13] Huang W., Ling S., Li C., Omenetto F.G., Kaplan D.L. (2018). Silkworm silk-based materials and devices generated using bio-nanotechnology. Chem. Soc. Rev..

[14] Kim S.H., Yeon Y.K., Lee J.M., Chao J.R., Lee Y.J., Seo Y.B., Sultan M.T., Lee O.J., Lee J.S., Yoon S.-I. (2018). Precisely printable and biocompatible silk fibroin bioink for digital light processing 3D printing. Nat. Commun..

[15] Rockwood D.N., Preda R.C., Yucel T., Wang X., Lovett M.L., Kaplan D.L. (2011). Materials fabrication from Bombyx mori silk fibroin. Nat. Protoc..

[16] Roh T.T., Chen Y., Paul H.T., Guo C., Kaplan D.L. (2019). 3D bioengineered tissue model of the large intestine to study inflammatory bowel disease. Biomaterials.

[17] Vieira M.G.A., da Silva M.A., dos Santos L.O., Beppu M.M. (2011). Natural-based plasticizers and biopolymer films: A review. Eur. Polym. J..

[18] Bocqué M., Voirin C., Lapinte V., Caillol S., Robin J.J. (2016). Petro-based and bio-based plasticizers: Chemical structures to plasticizing properties. J. Polym. Sci. Part A Polym. Chem..

[19] Mekonnen T., Mussone P., Khalil H., Bressler D. (2013). Progress in bio-based plastics and plasticizing modifications. J. Mater. Chem. A.

[20] Jose R.R., Brown J.E., Polido K.E., Omenetto F.G., Kaplan D.L. (2015). Polyol-Silk Bioink Formulations as Two-Part Room-Temperature Curable Materials for 3D Printing. ACS Biomater. Sci. Eng..

[21] Lu S., Wang X., Lu Q., Zhang X., Kluge J.A., Uppal N., Omenetto F., Kaplan D.L. (2010). Insoluble and flexible silk films containing glycerol. Biomacromolecules.

[22] Brown J.E., Davidowski S.K., Xu D., Cebe P., Onofrei D., Holland G.P., Kaplan D.L. (2016). Thermal and Structural Properties of Silk Biomaterials Plasticized by Glycerol. Biomacromolecules.

[23] Jiang C.Y., Wang X.Y., Gunawidjaja R., Lin Y.H., Gupta M.K., Kaplan D.L., Naik R.R., Tsukruk V.V. (2007). Mechanical properties of robust ultrathin silk fibroin films. Adv. Funct. Mater..

[24] Wang Y., Zheng Z., Cheng Q., Kaplan D.L., Li G., Wang X. (2020). Ductility and Porosity of Silk Fibroin Films by Blending with Glycerol/Polyethylene Glycol and Adjusting the Drying Temperature. ACS Biomater. Sci. Eng..

[25] Le Tien C., Letendre M., Ispas-Szabo P., Mateescu M., Delmas-Patterson G., Yu H.-L., Lacroix M. (2000). Development of biodegradable films from whey proteins by cross-linking and entrapment in cellulose. J. Agric. Food Chem..

[26] Bergo P., Sobral P. (2007). Effects of plasticizer on physical properties of pigskin gelatin films. Food Hydrocoll..

[27] Guerrero P., De la Caba K. (2010). Thermal and mechanical properties of soy protein films processed at different pH by compression. J. Food Eng..

[28] Barth A. (2007). Infrared spectroscopy of proteins. Biochim. Biophys. Acta.

[29] Lu Q., Hu X., Wang X., Kluge J.A., Lu S., Cebe P., Kaplan D.L. (2010). Water-insoluble silk films with silk I structure. Acta Biomater..

[30] Asakura T., Suzuki Y., Nakazawa Y., Holland G.P., Yarger J.L. (2013). Elucidating silk structure using solid-state NMR. Soft Matter..

[31] Hu X., Kaplan D., Cebe P. (2007). Effect of water on the thermal properties of silk fibroin. Thermochim. Acta.

[32] Hu X., Kaplan D., Cebe P. (2006). Determining beta-sheet crystallinity in fibrous proteins by thermal analysis and infrared spectroscopy. Macromolecules.

[33] Kim D.H., Kim Y.S., Amsden J., Panilaitis B., Kaplan D.L., Omenetto F.G., Zakin M.R., Rogers J.A. (2009). Silicon electronics on silk as a path to bioresorbable, implantable devices. Appl. Phys. Lett..

[34] Yazawa K., Ishida K., Masunaga H., Hikima T., Numata K. (2016). Influence of Water Content on the beta-Sheet Formation, Thermal Stability, Water Removal, and Mechanical Properties of Silk Materials. Biomacromolecules.

[35] Lawrence B.D., Wharram S., Kluge J.A., Leisk G.G., Omenetto F.G., Rosenblatt M.I., Kaplan D.L. (2010). Effect of hydration on silk film material properties. Macromol. Biosci..

