# Supplementary material for: Processing-Structure-Properties Relationships of Glycerol-Plasticized Silk Films
Source: Molecules. 2022 Feb 16;27(4):1339. doi: 10.3390/molecules27041339 (PMC8877885; doi:10.3390/molecules27041339)
Supplement: Supplementary file 1 [file molecules-27-01339-s001.zip › molecules-1571423-supplementary.pdf]

Supporting Information

Processing-Structure-Properties Relationships of Glycerol-Plasticized Silk Films

Hao Lyu [1,2], Ziyang Sun [2], Yang Liu [3], Xin Yu [2,*] and Chengchen Guo [2,*]

[1] School of Materials Science and Engineering, Zhejiang University, Hangzhou, 310027, China
[2] School of Engineering, Westlake University, Hangzhou, Zhejiang Province, 310024, China
[3] Institute of Biomedical Engineering and Health Sciences, Changzhou University, Changzhou, Jiangsu, 213164, China
* Correspondence: yuxin@westlake.edu.cn; guochengchen@westlake.edu.cn

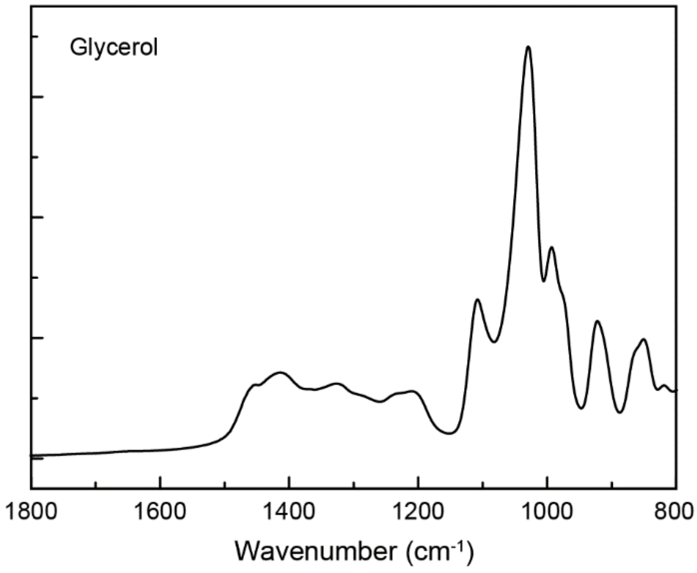

Figure S1. FTIR spectra of pure glycerol in the range of 800-1800 cm^{-1}.

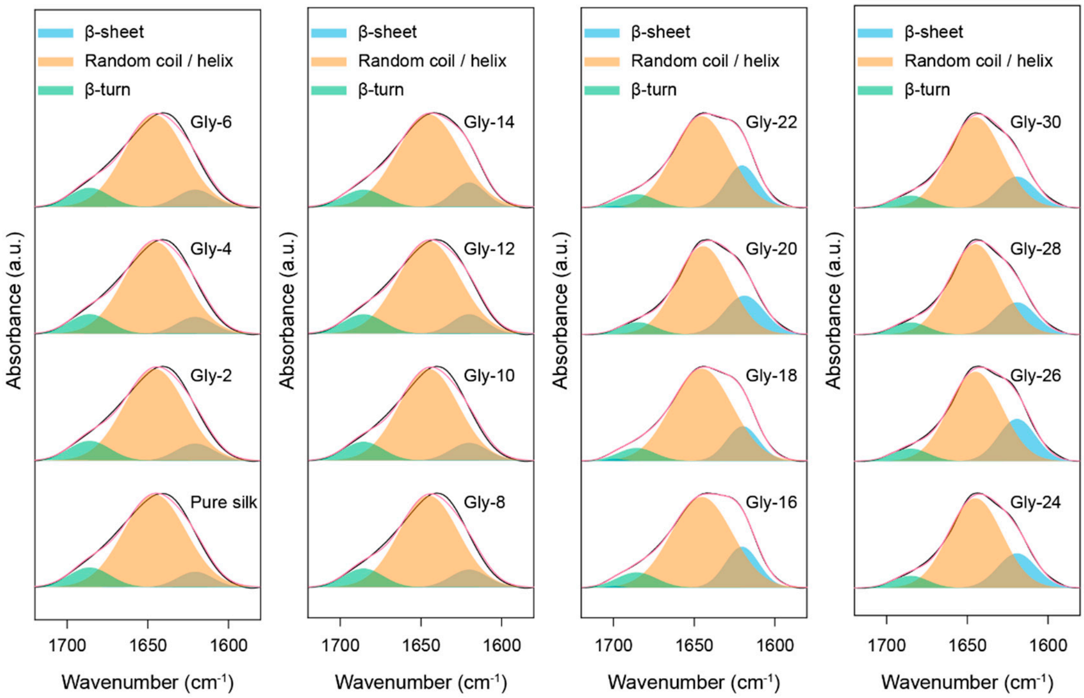

Figure S2. The deconvolution of the amide I region for glycerol-plasticized silk films.

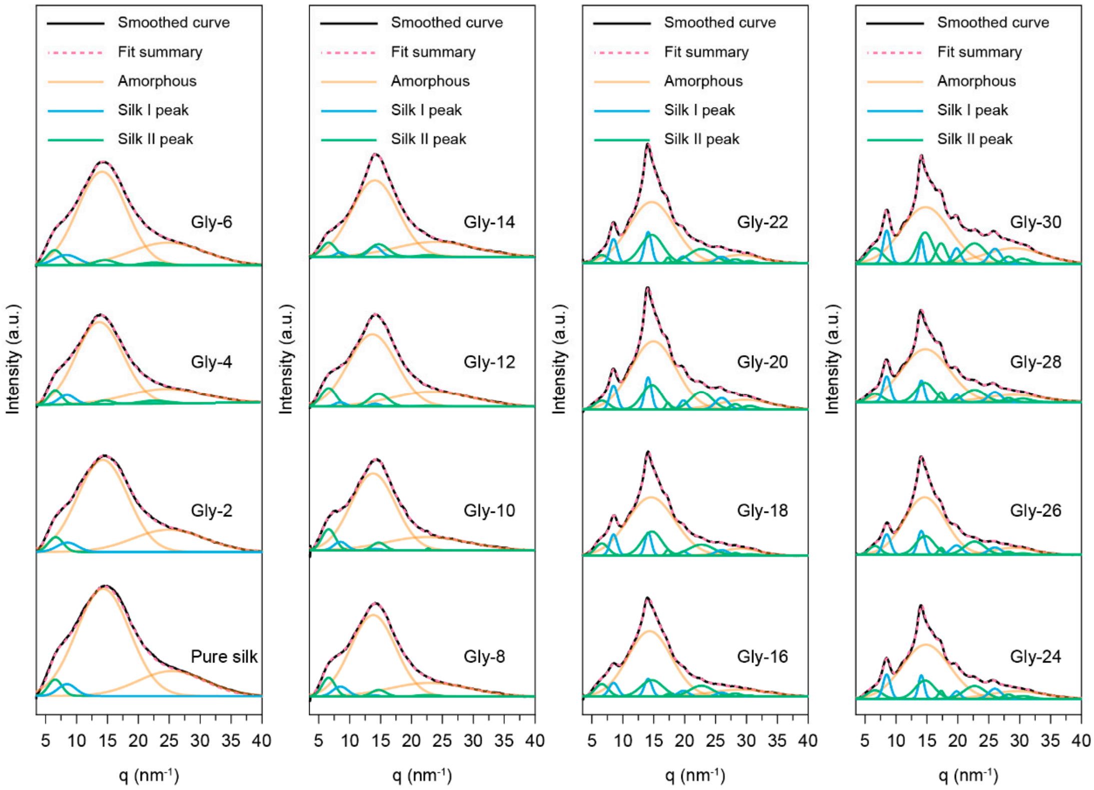

Figure S3. The deconvolution and peak fitting process of XRD curve for different silk-glycerol films. The peaks used in the deconvolution include crystalline components (6.6 nm^{-1}, 8.5 nm^{-1}, 14.1 nm^{-1}, 14.7 nm^{-1}, 17.3 nm^{-1}, 19.8 nm^{-1}, 22.7 nm^{-1}, 26 nm^{-1}, 28.1 nm^{-1}, 30.5 nm^{-1}) and amorphous components (~15.0 nm^{-1}, ~28.0 nm^{-1}).

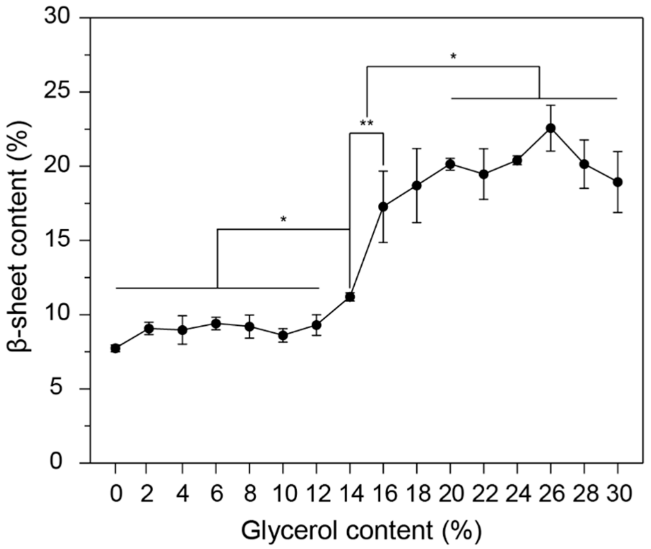

Figure S4. Statistical analysis for β-sheet contents of the glycerol-plasticized silk films. * Significant differences between groups ($P < 0.05$). ** Very significant differences between groups ($P < 0.01$). Data represent the AV±SD (n=3).
